# Tanshinone IIA potentiates the therapeutic efficacy of glucocorticoids in lipopolysaccharide-treated HEI-OC1 cells through modulation of the FOXP3/Nrf2 signaling pathway: TA enhances GC efficacy in LPS-induced HEI-OC1 cells via FOXP3/Nrf2 axis

**DOI:** 10.3724/abbs.2024194

**Published:** 2024-11-01

**Authors:** Jie Li, Xiaoyan Zhu, Shiming Ye, Qi Dong, Jie Hou, Jing Liu, Wandong She

**Affiliations:** 1 Department of Otolaryngology-Head and Neck Surgery Nanjing Drum Tower Hospital Nanjing Drum Tower Hospital Clinical College of Nanjing University of Chinese Medicine Nanjing University of Chinese Medicine Nanjing 210008 China; 2 Department of Otolaryngology Nantong Hospital Affiliated to Nanjing University of Chinese Medicine Nantong 226000 China; 3 Department of Otolaryngology-Head and Neck Surgery . Nanjing Drum Tower Hospital Affiliated Hospital of Medical School Nanjing University Nanjing 210008 China; 4 Otorhinolaryngology Research Institute of Nanjing Drum Tower Hospital Nanjing 210008 China

**Keywords:** tanshinone IIA, sudden sensorineural hearing loss, glucocorticoid resistance, FOXP3

## Abstract

Glucocorticoids (GCs) are commonly used to treat sudden sensorineural hearing loss (SSNHL), although some patients are resistant to this therapeutic approach. Clinical studies have demonstrated the efficacy of tanshinone IIA (TA) in combination with GC for managing various human ailments. However, it remains unclear whether TA can mitigate GC resistance in SSNHL. Our aim is to elucidate the role of NRF2-induced transcriptional regulation of HDAC2 in influencing GC resistance and investigate the involvement of TA-related molecular pathways in GC resistance. Here, HEI-OC1 cells are treated with lipopolysaccharide (LPS) to establish an *in vitro* model for SSNHL. The cells are subsequently treated with dexamethasone (DXE) or DXE + TA. RT-qPCR and western blot analysis are used to measure the mRNA and protein levels of Forkhead box P3 (FOXP3), nuclear factor erythroid 2-related factor 2 (NRF2), and histone deacetylase 2 (HDAC2). Cell Counting Kit-8 (CCK-8) and 5-ethynyl-2’-deoxyuridine (EdU) assays are carried out to assess cell proliferation. Flow cytometry analysis is performed to evaluate apoptosis. Mechanistic studies involve chromatin immunoprecipitation (ChIP), luciferase reporter, and DNA pull-down assays. Our results show that treatment with TA + DEX significantly increases proliferation and suppresses apoptosis in LPS-treated HEI-treated OC1 cells. TA upregulates HDAC2 expression by activating NRF2-mediated transcription of HDAC2, with the NRF2-HDAC2 binding site located at bases 419–429 (ATGACACTCCA) in the promoter sequence of *HDAC2*. Furthermore, TA upregulates FOXP3 expression to activate NRF2 transcription, with the predicted FOXP3-binding site located at bases 864–870 (GCAAACA) in the promoter sequence of *NRF2*. In summary, these findings suggest that TA enhances the therapeutic effects of GC on the proliferation and apoptosis of HEI OC1 cells by increasing FOXP3/Nrf2 expression. These results indicate that TA may be promising for ameliorating GC resistance in patients with SSNHL.

## Introduction

Sudden sensorineural hearing loss (SSHL) is a prevalent otological emergency. Previous studies have shown that SSHL affects 5–27 per 100,000 people per year, with nearly 66,000 new cases annually in the United States of America [1]. Sensory hearing loss often occurs due to damaged or deficient cochlear hair cells; hair cells may be abnormal at birth or damaged during an individual’s lifetime. The proposed etiologies of primary SSHL include virus infection, vascular insufficiency, autoimmune disorders, and stress theory [ 2, 3] . There is no proven or recommended treatment or cure for SSHL. The treatment of SSHL remains one of the most challenging issues in contemporary otorhinolaryngology. Therefore, it is necessary to explore promising therapeutic strategies for patients with SSHL. HEI-OC1 is one of the few mouse auditory cell lines available for research. Originally proposed as an *in vitro* system for screening ototoxic drugs, these cells have been used to investigate drug-activated apoptotic pathways, autophagy, senescence, mechanisms of cell protection, inflammatory responses, cell differentiation, genetic and epigenetic effects of pharmacological drugs, effects of hypoxia, oxidative and endoplasmic reticulum stress, and the expressions of molecular channels and receptors [4]. Currently, the HEI-OC1 cell line is widely used in the field of hair cell research. To a certain extent, it can be used to speculate on *in vivo* changes and establish a hair cell injury and inflammation model.

Currently, glucocorticoids (GCs) are widely used to treat SSHL and various immune and inflammatory diseases [ 5– 8] . Although most patients with SSHL respond well to GCs, approximately 20% of patients show no significant hearing improvement after GC treatment, indicating GC resistance in these patients [ 9, 10] .

Fortunately, traditional Chinese medicine has been combined with Western medicine to prevent and reverse drug resistance in cancer cells [11]. This approach has also been applied to the treatment of SSHL. For example, treating SSHL patients with a combination of GCs and breviscapine (a traditional Chinese medicine) has been proven to be more effective than using GCs alone [12]. Therefore, exploring the potential of traditional Chinese medicine for preventing and reversing GC resistance in SSHL is worthwhile.

Tanshinone IIA (TA) is a bioactive compound derived from the rhizomes and roots of *Salvia miltiorrhiza* Bunge, commonly known as Danshen, a traditional Chinese herb. TA has garnered attention for its therapeutic potential in various diseases, including liver disorders [13], atherosclerosis, cancers, and other diseases [ 14– 16] . At the molecular level, TA influences various pathways. For example, it attenuates neuroinflammation by suppressing the RAGE/NF-κB signaling pathway, thereby potentially impeding the progression of Alzheimer’s disease [17]. Moreover, TA protects hair cells against radiation-induced damage by suppressing p65/NF-κB nuclear translocation and modulating the p53/p21 signaling pathway [18]. Furthermore, TA sodium sulfonate injection has shown promise in enhancing hearing and improving the hemorheological parameters and immune functions of SSHL patients. However, the molecular mechanisms underlying TA in SSHL remain to be explored.

Recent research has proposed a correlation between diminished nuclear factor erythroid 2-related factor 2 (NRF2) and histone deacetylase 2 (HDAC2) protein levels and GC resistance in SSHL patients [ 10, 19] . Our previous studies revealed decreased expressions of HDAC2 and the apoptosis-inhibiting genes *BCL-2* and *BCL-xL* in an LPS-induced hearing loss animal model and an LPS-treated HEI-OC1 cell injury model [20]. Furthermore, elevated NRF2 expression mitigates GC insensitivity by increasing HDAC2 level *in vitro* [ 21, 22] .

Forkhead box P3 (FOXP3) is a pivotal transcription factor that regulates the expression of its target RNAs [23]. However, its involvement in SSHL remains largely unexplored.

In the present study, we applied bioinformatics tools to analyze the relationship between FOXP3 and NRF2 in the HEI-OC1 cell injury model. We focused on the role of NRF2-mediated transcriptional modulation of HDAC2 to elucidate the impact of TA-related molecular pathways on GC resistance. Our results showed that the combination of TA and DEX can significantly improve LPS-induced hair cell damage, which provides a new therapeutic strategy for the clinical treatment of SSHL.

## Materials and Methods

### Cell culture and treatment

The HEI-OC1 cell line obtained from Professor Chai Renjie’s Laboratory (Southeast University Life Science Research Institute, Nanjing, China) was maintained in DMEM supplemented with 10% fetal bovine serum (FBS) and 100 U/mL penicillin. Another cell line, 293T, purchased from ATCC (Manassas, USA), was maintained in DMEM supplemented with 10% FBS and 2 mM glutamine.

As previously described, the HEI-OC1 cell injury model was induced by treatment with 0.1 μg/mL LPS (MCE, Monmouth Junction, USA) for 24 h [24]. To detect the effect of dexamethasone (DEX) on LPS-induced apoptosis in HEI-OC1 cells, 50 μg/mL dexamethasone (DEX) was used to treat the cells as previously described [25]. TA purchased from MCE was used to treat the cells, along with 10 μM DEX, which can significantly inhibit LPS-induced inflammation as previously described [26], in the present study.

### Plasmid transfection

Three small interfering RNAs (siRNAs) targeting NRF2 (si-NRF2-1, si-NRF2-2, and si-NRF2-3) or HDAC2 (si-HDAC2-1, si-HDAC2-2, and si-HDAC2-3) were synthesized by GenePharma (Shanghai, China) for the silencing of each target, and their sequences are shown in Table 1. Nontargeted random siRNA was used as the negative controls (si-NCs). For the overexpression of NRF2 or FOXP3, the full-length sequence of each target gene was separately subcloned and inserted into a pcDNA3.1 expression vector (GenePharma), while the pcDNA3.1 empty vector was used as the control. The plasmids were transfected into HEI-OC1 cells using Lipofectamine 2000 (Invitrogen, Carlsbad, USA). The cells were harvested for subsequent experiments after they were cultured for 48 h.

**Table:** **Table 1** Sequences of siRNAs used in this study

siRNA	Sequence (5′→3′)
si-NC1	AATTCTCCGAACGTGTCACGT
si-NRF2-1	TATGTCAATCAAATCCATGTC
si-NRF2-2	CATTGATGTTTCTGATCTATC
si-NRF2-3	TCATCTAGTTGTAACTGAGCG
si-NC2	GGTTTACATGTTGTGTGA
si-HDAC2-1	GGTCAATAAGACCAGATAACA
si-HDAC2-2	TATACTCAGACATGTTATCTG
si-HDAC2-3	TCTATACCATCTCTCATTGGA

### Real-time quantitative polymerase chain reaction (RT-qPCR)

Total RNA was isolated using Trizol reagent (Sangon, Shanghai, China). The integrity and purity of the extracted total RNA were measured using a NanoDrop One ultramicro-UV spectrophotometer (Thermo Fisher Scientific, Waltham, USA). A total of 2 μg of RNA was used for reverse transcription. M-MLV reverse transcriptase (Sigma-Aldrich, St Louis, USA) was used to reverse transcribe isolated RNA into cDNA. Reverse transcription was conducted at 25°C for 5 min, 42°C for 60 min, and 70°C for 5 min. PCR was then performed using SYBR Green PCR Master Mix (Takara, Kyoto, Japan) for 40 cycles (95°C for 3 s and 60°C for 30 s). Relative RNA levels were calculated via the 2 ^–ΔΔCt^ method by normalization to *GAPDH*. The sequences of primers are shown in Table 2 **.**

**Table:** **Table 2** Sequences of primers used in this study

Gene	Primer sequence (5′→3′)
*NRF2*	F: GGTTGCCCACATTCCCAAAT R: AGCAATGAAGACTGGGCTCT
*HDAC2*	F: TGGTGTCCAGATGCAAGCTA R: GCCACATTTCTTCGACCTCC
*FOXP3*	F: CCCGGATGTGAGAAGGTCTT R: CTTGTCGGATGATGCCACAG
*GAPDH*	F: CTCAGACACCATGGGGAAGGTGA R: ATGATCTT2GAGGCTGTTGTCATA

### Western blot analysis

Total protein was extracted from HEI-OC1 cells using RIPA lysis buffer. The supernatant was collected after centrifugation at 10,000 *g* for 10 min at 4°C.A BCA protein assay kit (Pierce, Rockford, USA) was used to determine the protein concentration. Proteins were separated by 10% SDS-PAGE and transferred onto PVDF membranes (Millipore, Billerica, USA). The membranes were blocked with 5% nonfat milk in TBST for 1 h and then incubated with one of the following primary antibodies: anti-BCL-2 (1:2000; Proteintech Group, Inc., Rosemont, USA), anti-BAX (1:2000, Proteintech), anti-GR (1:20,000; Proteintech), anti-NRF2 (1:1000; Proteintech), anti-HDAC2 (1:20,000; Proteintech), anti-FOXP3 (1:1000; Proteintech) or anti-β-actin (1:20,000; Proteintech) at 4°C overnight. After being washed four times with TBST, the membranes were incubated with a mouse-derived secondary antibody: anti-IgG (1:2000; Proteintech) at room temperature for 2 h. Protein blots were visualized on an enhanced chemiluminescence (ECL) system (Tanon, Shanghai, China). β-Actin was used as an internal reference. Image-Pro Plus 6.0 was used for density quantification of all protein bands.

### Cell counting kit-8 (CCK-8) assay

For the CCK-8 assay, stably transfected cells were seeded into 96-well plates at 3000 cells per well. After 24 h of incubation for attachment, the cells were incubated with fresh medium supplemented with LPS, DEX, or DEX + TA for 0, 24, 48, or 72 h. Subsequently, the cells were incubated with 10 μL of CCK-8 solution (Beyotime, Shanghai, China) at 37 °C for another 2 h. The absorbance value at 450 nm was measured with an EnVision® multimode plate reader (Perkin Elmer, Waltham, USA).

### 5-Ethynyl-2′-deoxyuridine (EdU) assay

Cell proliferation was assessed via an EdU assay kit (RiboBio, Guangzhou, China). In brief, the transfected cells were seeded into 96-well plates and incubated with EdU at 37°C for 2 h. The cells were washed with PBS and fixed in 4% paraformaldehyde (Sangon Biotech) at 4°C for 30 min. The nuclei were stained with 400 μL of DAPI (Sigma-Aldrich) at room temperature for 30 min. Cell proliferation was observed using a Leica fluorescence microscope (200×; Leica, Wetzlar, Germany). The percentage of EdU-positive cells in five randomly selected fields was calculated using ImageJ software v1.8.0 (National Institutes of Health, Bethesda, USA).

### Apoptosis assay

According to the instructions, cell apoptosis was detected using an Annexin-V-FITC and propidium iodide (PI) apoptosis detection kit (BD Biosciences, Franklin Lakes, USA). Briefly, the cells were collected and washed twice in cold PBS. The cells were then fixed with 4% paraformaldehyde (Sangon Biotech) at –20°C overnight, suspended in 600 μL of eBioscience™ flow cytometry binding buffer (Thermo Fisher Scientific) at a concentration of 10 ^6^ cells/mL, and then stained with 5 μL of Annexin V/FITC and 5 μL of propidium iodide (BD Biosciences) in the dark for 15 min. Flow cytometry was used to assess the number of apoptotic cells in triplicate (FACS Calibur; Becton Dickinson, Franklin Lakes, USA). The results were analyzed using FlowJo 7.6 software (Tree Star, Inc., Ashland, USA).

### Chromatin immunoprecipitation (ChIP) assay

A Magna ChIP™ RNA-Binding Protein Immunoprecipitation kit (Millipore) was used for the ChIP assay. Briefly, the cells were cross-linked with 1% formaldehyde for 10 min and sonicated to shear the DNA into 200–1000 base pairs. The cell lysates were incubated with protein A/G beads coated with anti-NRF2 or anti-FOXP3 antibody (Proteintech) at 4°C overnight. Anti-rabbit IgG was used as a negative control. After washing, the bead-bound immunocomplexes were eluted with elution buffer. To decrosslink the DNA-protein complex, 5 M NaCl was added to the samples, which were subsequently heated at 65°C for 4 h, treated with proteinase K, and further incubated at 45°C for 1 h. Finally, the immunoprecipitated DNA was purified for RT-qPCR analysis.

### Luciferase reporter assay

The whole sequence of the *HDAC2* promoter with or without *HDAC2* [named wild type (WT) and mutant type (MUT)] was inserted into the pGL3 luciferase vector to generate pGL3-HDAC2 promoter-WT or pGL3-HDAC2 promoter-MUT. Similarly, the entire sequence of the *NRF2* promoter with or without *FOXP3* was inserted into the pGL3 luciferase vector (Promega, Madison, USA) to generate pGL3-NRF2 promoter-WT or pGL3-NRF2 promoter-MUT. To demonstrate the functional sites, the pGL3-HDAC2 promoter-WT and pGL3-HDAC2 promoter-MUT were co-transfected with the pcDNA3.1-NRF2 or pcDNA3.1 empty vector into 293T cells. Similarly, the pGL3-NRF2 promoter-WT and pGL3-NRF2 promoter-MUT vectors were co-transfected with the pcDNA3.1-FOXP3 or pcDNA3.1 empty vector into 293T cells. Transfections were completed via the use of Lipofectamine 2000 Transcription Reagent (Thermo Fisher Scientific) at 37°C for 46 h. Finally, the firefly luciferase activity was detected via normalization to the *Renilla* luciferase activity using a Luciferase Reporter Assay System (Promega).

### DNA pull-down assay

Biotinylated wild-type or mutated *HDAC2* promoter/ *NRF2* promoter probes (Bio-HDAC2 promoter-WT/MUT or Bio-NRF2 promoter-WT/MUT) or NC probes (Bio-NC) synthesized by RiboBio were added to 1% Triton X-100, followed by heating at 95°C for 2 min and cooling in an ice bath for 3 min. The denatured biotinylated RNA was mixed with 15 μL of streptavidin beads (Thermo Fisher Scientific) to pull down the biotinylated protein-DNA complex. The DNA-bead complexes were incubated with 100 mL of cell lysate obtained via RIPA lysis buffer. Finally, the proteins eluted from the DNA were subjected to western blot analysis.

### Statistical analysis

Data are shown as the mean ± standard deviation (SD) from three independent experiments. and were plotted via GraphPad Prism v8.0 (GraphPad Software, San Diego, USA). SPSS V22.0 software (IBM, Armonk, USA) was used for statistical analysis of all the data. Differences between two groups were compared via an unpaired Student’s *t* test. Differences among multiple groups were compared via one-way analysis of variance (ANOVA) with Dunnett’s or Tukey’s *post hoc* test. *P*  < 0.05 indicates that the difference is statistically significant.

## Results

### TA enhances HDAC2 protein level in LPS-treated HEI-OC1 cells

**Figure 1:**
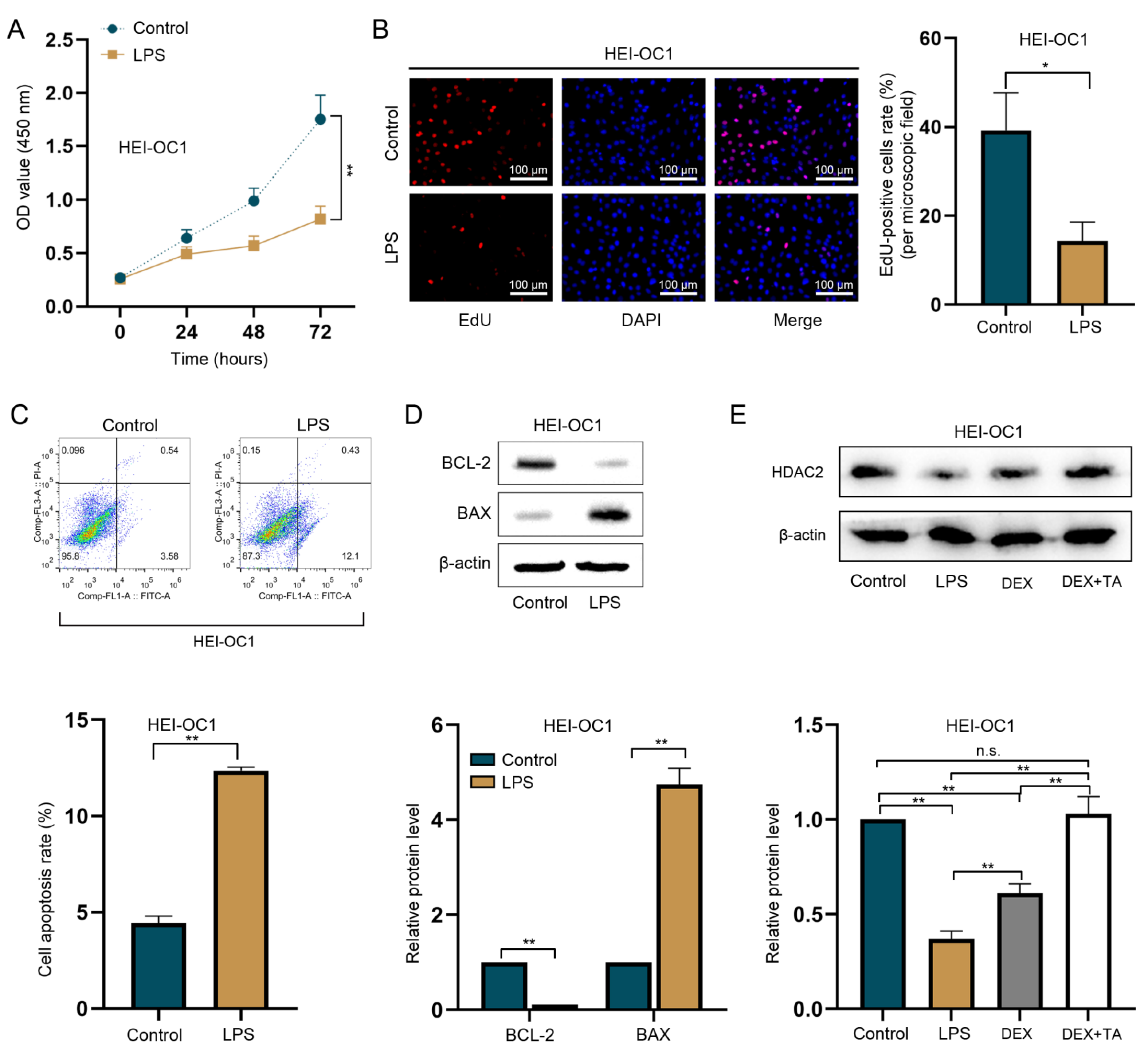
Figure 1 TA enhances the HDAC2 protein level in HEI-OC1 cells treated with LPS (A,B) CCK-8 and EdU assays were used to detect the proliferation of HEI-OC1 cells treated with LPS. As detected by CCK-8 and EdU assays, cell proliferation was markedly suppressed by LPS treatment compared with that in the control group. Scale bar: 100 μm. (C) Apoptosis in HEI-OC1 cells treated with LPS was assessed by flow cytometry analysis. The percentage of apoptotic cells was increased significantly by LPS treatment. (D) Western blot analysis of BCL-2 and BAX expressions in HEI-OC1 cells treated with LPS. The BAX protein level increased, whereas the BCL-2 protein level decreased after LPS treatment. (E) Western blot analysis of HDAC2 protein expression in HEI-OC1 cells treated with LPS, DEX, or DEX + TA. HDAC2 protein level was decreased in HEI-OC1 cells treated with LPS alone, while its level was partly reversed by DEX treatment and completely reversed by treatment with both DEX and TA. *P < 0.05 and **P < 0.01. n.s., not significant.

In the present study, we utilized an *in vitro* LPS injury model [20] to investigate the impact of TA on GC sensitivity in SSHL. We conducted proliferation and apoptosis assays on HEI-OC1 cells treated with 0.1 μg/mL LPS for 24–72 h. Cell proliferation, as assessed via CCK-8 and EdU assays, was markedly lower in the LPS-treated group than in the control group ( *P*  < 0.01; Figure 1A,B). Moreover, LPS treatment markedly increased the percentage of apoptotic cells ( *P*  < 0.01; Figure 1C), which was further confirmed by elevated BAX protein level and reduced BCL-2 protein level ( Figure 1D). Additionally, we evaluated HDAC2 protein expression in HEI-OC1 cells treated with LPS, LPS + DEX, or LPS + DEX + TA. HDAC2 protein level was reduced in HEI-OC1 cells treated with LPS alone ( *P*  < 0.01; Figure 1E), whereas this reduction was partially reversed by DEX treatment and further restored by combined treatment with DEX and TA ( *P*  < 0.01; Figure 1E).

### TA enhances proliferation and GR expression and inhibits apoptosis in LPS-treated HEI-OC1 cells

**Figure 2:**
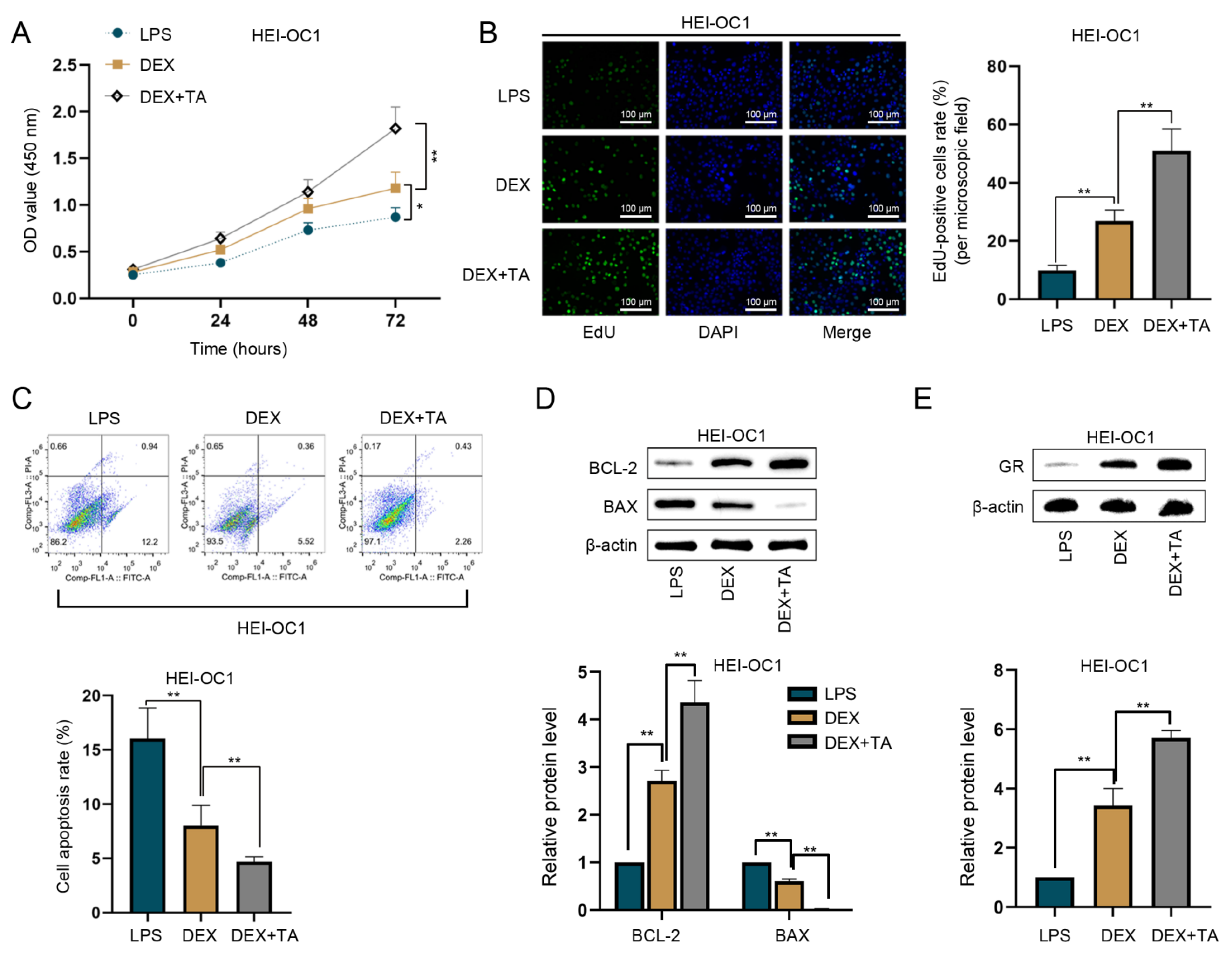
Figure 2 TA increases GR level and proliferation and inhibits apoptosis in HEI-treated OC1 cells (A,B) CCK-8 and EdU assays were used to detect the proliferation of LPS-treated HEI-OC1 cells. The results revealed that 50 μg/mL DEX increased the proliferation rate of HEI-OC1 cells treated with LPS, whereas DEX + TA (10 μM) increased the proliferation rate of HEI-OC1 cells treated with LPS. Scale bar: 100 μm. (C) Apoptosis induced by LPS in HEI-OC1 cells was assessed by flow cytometry analysis. DEX inhibited the apoptosis induced by LPS in HEI-OC1 cells and the additional TA treatment strongly suppressed apoptosis compared with DEX alone. (D) Western blot analysis of BCL-2 and BAX expressions in HEI-OC1 cells treated with LPS. DEX alone increased BCL-2 protein level and decreased Bax protein level whereas TA significantly enhanced the inhibitory effect of DEX on cell apoptosis. (E) Western blot analysis of GR expression in HEI-OC1 cells. Compared with that in HEI-OC1 cells treated with DEX alone, the GR protein level in HEI-OC1 cells treated with LPS was elevated after TA treatment. *P < 0.05 and **P < 0.01.

We then evaluated the effects of TA on the proliferation and apoptosis of LPS-treated HEI-OC1 cells. Treatment with 50 μg/mL DEX significantly increased the proliferation rate of HEI-OC1 cells ( *P*  < 0.05), whereas combined treatment with DEX (50 μg/mL) + TA (10 μM) increased the proliferation rate of HEI-OC1 cells ( *P*  < 0.01; Figure 2A,B). Moreover, DEX inhibited the LPS-induced apoptosis of HEI-OC1 cells ( *P*  < 0.01), and compared with DEX alone, TA further suppressed apoptosis more effectively ( *P*  < 0.01; Figure 2C). Western blot analysis results further supported these findings, showing that DEX increased BCL-2 protein level and decreased BAX protein level, with TA significantly enhancing the antiapoptotic effects of DEX ( Figure 2D).

Previous work revealed that GR mediates the physiologic and pharmacologic actions of GCs [9]. Consequently, we investigated the impact of TA on GR expression. Western blot analysis revealed that the GR protein level was elevated after TA treatment compared with DEX treatment alone in LPS-treated HEI-OC1 cells ( *P*  < 0.01; Figure 2E). Previous studies suggested that increased GR and HDAC2 levels and enhanced cell proliferation are associated with reduced GC resistance [ 6, 9, 10] . Therefore, TA can potentially inhibit GC resistance by upregulating GR and HDAC2 expression, promoting proliferation, and inhibiting apoptosis.

### TA enhances HDAC2 expression through NRF2-mediated transcription activation

Recent research has demonstrated low NRF2 and HDAC2 expression levels in patients with refractory SSHL [10]. Our previous study also indicated that reduced NRF2, HDAC2, and GR expression levels are associated with decreased GC sensitivity in SSHL patients [ 9, 10, 19] . HDAC2 is a critical component of the GC-GR complex, which mediates the trans-repression of NF-кB transcriptional activity by deacetylating histones in proinflammatory genes and deacetylating GR [9]. We hypothesized that TA might regulate NRF2 and HDAC2 expression, thereby influencing GC sensitivity in SSHL patients.

**Figure 3:**
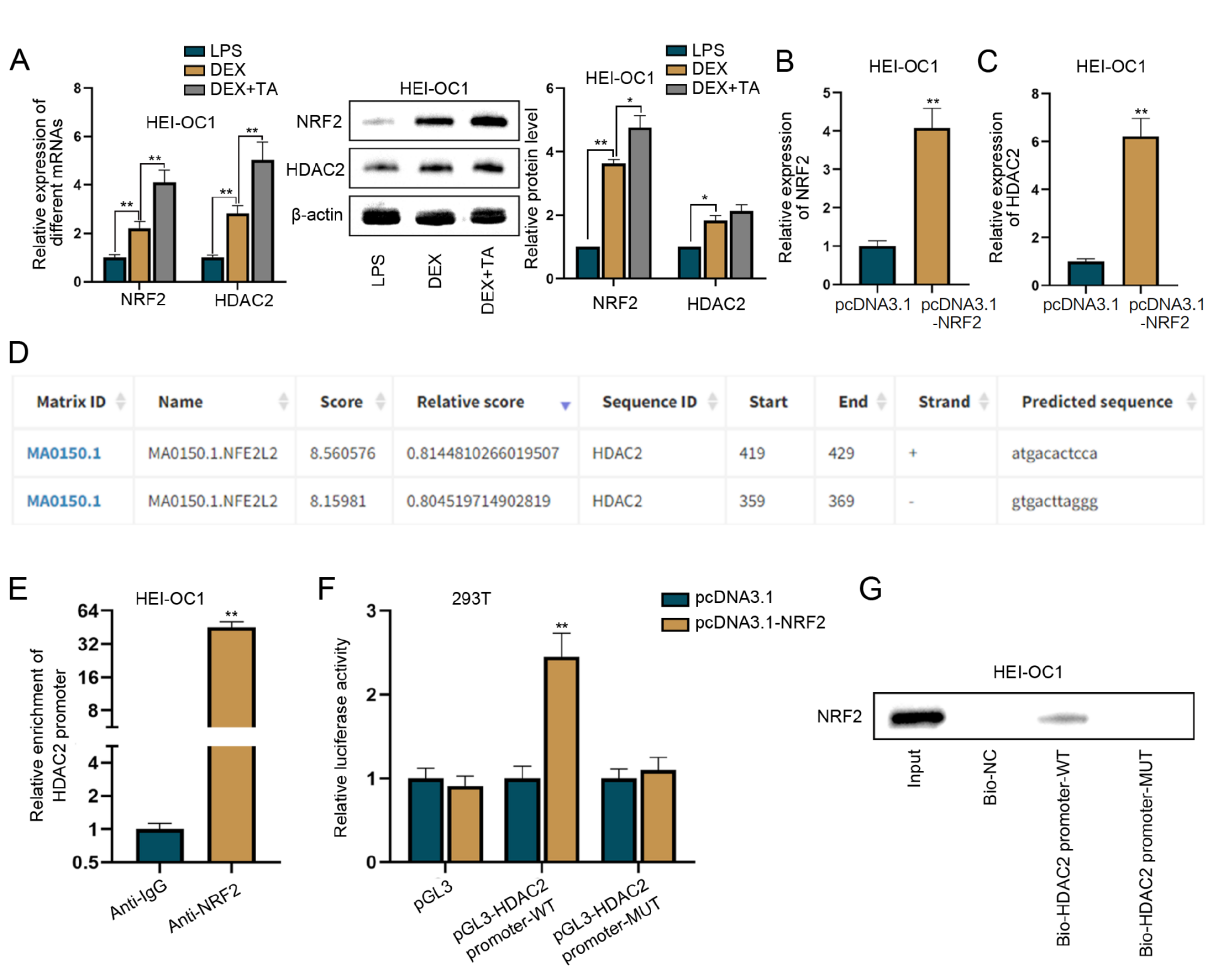
Figure 3 TA enhances HDAC2 expression through NRF2-mediated transcription activation (A) RT-qPCR and western blot analysis of NRF2 and HDAC2 expressions in HEI-OC1 cells treated with LPS, DEX or DEX+TA. TA increased NRF2 and HDAC2 mRNA and protein levels in DEX-treated HEI-OC1 cells. (B) RT-qPCR was used to assess the gene overexpression of NRF2 in HEI-OC1 cells. NRF2 was successfully overexpressed in HEI-OC1 cells. (C) HDAC2 mRNA levels were detected by RT-qPCR in HEI-OC1 cells overexpressing NRF2. We detected increased HDAC2 mRNA level in HEI-OC1 cells overexpressing NRF2. (D) A potential NRF2-binding site in the sequence of the HDAC2 promoter was predicted using the JASPAR database. The NRF2-binding site with the highest prediction score is located at bases 419–429 (ATGACACTCCA) in the HDAC2 promoter sequence. (E) ChIP detected enrichment of the HDAC2 promoter in immunoprecipitates immunoprecipitated with anti-NRF2. ChIP assay revealed abundant enrichment of the HDAC2 promoter in the immunoprecipitates with anti-NRF2, verifying the binding between NRF2 and the HDAC2 promoter. (F) Luciferase activity of different reporter constructs in NRF2-overexpressing 293T cells was detected. (G) DNA pull-down assay followed by western blot analysis was used to detect NRF2 expression in the complex pulled down by the Bio-HDAC2 promoter-WT. NRF2 overexpression increased the luciferase activity of the HDAC2 promoter-WT but did not affect the activity of the HDAC2 promoter-MUT (mutated sequence: TACTGTGAGGT at bases 419–429). *P < 0.05 and **P < 0.01.

We observed that TA further increased NRF2 and HDAC2 mRNA and protein levels in DEX-treated HEI-OC1 cells ( *P*  < 0.01; Figure 3A), suggesting that TA could reverse GC resistance in SSHL via the upregulation of NRF2 and HDAC2 expression. Next, we explored the potential regulatory relationship between NRF2 and HDAC2. After confirming the high efficiency of NRF2 overexpression ( *P*  < 0.01; Figure 3B), we detected increased HDAC2 mRNA level in HEI-OC1 cells overexpressing NRF2 ( *P*  < 0.01; Figure 3C). Given the role of NRF2-mediated transcriptional regulation [27], we speculated that NRF2 might activate HDAC2 transcription to upregulate its expression.

Using the JASPAR database ( https://jaspar.genereg.net/), we identified putative NRF2-binding sites in the *HDAC2* promoter sequence, with the highest prediction score located at bases 419–429 (ATGACACTCCA) ( Figure 3D). The ChIP assay results revealed significant enrichment of the *HDAC2* promoter in immunoprecipitates with anti-NRF2 ( Figure 3E), verifying the binding between NRF2 and the *HDAC2* promoter. Luciferase reporter assay indicated that NRF2 overexpression increased the luciferase activity of the HDAC2 promoter-WT but did not affect the activity of the HDAC2 promoter-MUT (mutated sequence: TACTGTGAGGT at bases 419–429; Figure 3F). A DNA pull-down assay also revealed that NRF2 directly binds to the HDAC2 promoter-WT rather than the HDAC2 promoter-MUT ( Figure 3G). Taken together, these findings suggest that TA upregulates HDAC2 expression by activating NRF2-mediated HDAC2 transcription.

### TA upregulates FOXP3 expression to activate NRF2 transcription

**Figure 4:**
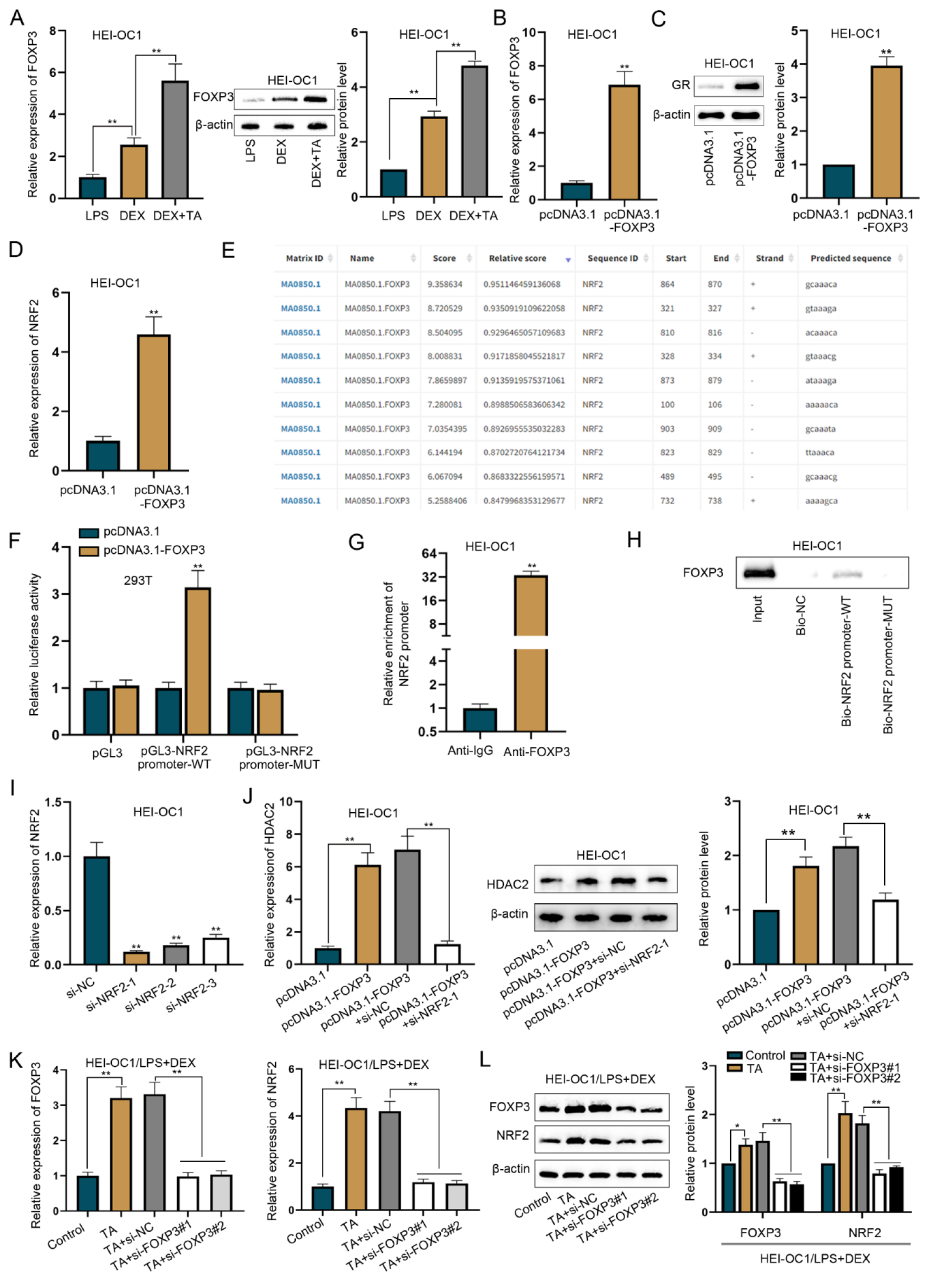
Figure 4 TA upregulates FOXP3 expression to activate NRF2 transcription (A) RT-qPCR and western blot analysis were used to measure FOXP3 expression in LPS-treated HEI-OC1 cells. FOXP3 mRNA and protein levels were increased in response to additional TA treatment. (B) The gene overexpression efficiency of FOXP3 in HEI-OC1 cells was tested via RT-qPCR. FOXP3 was overexpressed in HEI-OC1 cells. (C) GR expression in FOXP3-overexpressing HEI-OC1 cells was measured via western blot analysis. GR protein level was also increased in FOXP3-overexpressing HEI-OC1 cells. (D) NRF2 expression in FOXP3-overexpressing HEI-OC1 cells was measured by RT-qPCR. Elevated NRF2 mRNA levels were observed in FOXP3-overexpressing HEI-OC1 cells. (E) Potential FOXP3-binding sites in the NRF2 promoter sequence were predicted using the JASPAR database. According to the JASPAR database analysis, the predicted FOXP3-binding site with the highest prediction score is located at 864–870 bases (GCAAACA) in the NRF2 promoter sequence. (F) Luciferase activity of different reporter constructs in FOXP3-overexpressing 293T cells was detected by luciferase reporter assay. The luciferase activity of pGL3-NRF2 promoter-WT was increased by the upregulation of FOXP3 in 293T cells, whereas that of pGL3-NRF2 promoter-MUT (with a mutated sequence of CGTTTGT at amino acids 864–870) was not affected. (G) ChIP assay was performed to detect the physical binding between FOXP3 and the NRF2 promoter. ChIP assay revealed that the NRF2 promoter was highly enriched in the anti-FOXP3-precipitated complex. (H) A DNA pull-down assay followed by western blot analysis was used to detect FOXP3 expression in the complex pulled down by the Bio-NRF2 promoter-WT. DNA pull-down assay validated the effectiveness of the binding sites between FOXP3 and the NRF2 promoter. (I) The knockdown efficiency of the si-NRF2 plasmid was determined by RT-qPCR. All three siRNAs had high efficacy in terms of NRF2 knockdown. (J) HDAC2 expression in HEI-OC1 cells was measured by RT-qPCR and western blot analysis. The increased mRNA and protein levels of HDAC2 caused by FOXP3 overexpression were reversed by depletion of NRF2. (K) FOXP3 expression was downregulated in DEX-treated HEI OC1 cells. The NRF2 mRNA level was measured by RT-qPCR in DEX-treated HEI OC1 cells after FOXP3 knockdown. (L) Protein levels of FOXP3 and NRF2 were measured in DEX-treated HEI OC1 cells after FOXP3 knockdown. The NRF2 mRNA and protein levels were increased by TA treatment, whereas the levels were decreased by silencing of FOXP3. *P < 0.05 and **P < 0.01.

We subsequently investigated the underlying mechanism by which TA enhances NRF2 expression. Previous studies have shown that TA can increase FOXP3 expression to inhibit nasopharyngeal carcinoma progression [28]. By measuring FOXP3 expression in LPS-treated HEI-OC1 cells, we found that FOXP3 mRNA and protein levels were significantly increased in response to DEX treatment and further elevated with additional TA treatment ( *P*  < 0.01; Figure 4A). The transcription factor FOXP3 is expressed in various tissues and is crucial for antitumor and anti-inflammatory activities [29]. Hence, we speculated that TA-induced NRF2 upregulation might occur via FOXP3.

Upon successful overexpression of FOXP3 in HEI-OC1 cells ( *P*  < 0.01; Figure 4B), we observed that the GR protein level was also increased ( *P*  < 0.01; Figure 4C) and that the NRF2 mRNA level was elevated ( *P*  < 0.01; Figure 4D). According to the results of the JASPAR database analysis, the predicted FOXP3-binding site with the highest prediction score is located at bases 864–870 (GCAAACA) in the *NRF2* promoter sequence ( Figure 4E). Luciferase reporter assay revealed that the luciferase activity of the pGL3-NRF2 promoter-WT was increased by FOXP3 upregulation in 293T cells, whereas the activity of the pGL3-NRF2 promoter-MUT (mutated sequence: CGTTTGT at bases 864–870) was not affected ( Figure 4F). The results of the ChIP assay indicated that the *NRF2* promoter was highly enriched in the anti-FOXP3-precipitated complex ( Figure 4G). DNA pull-down assay also validated the binding sites between FOXP3 and the *NRF2* promoter ( Figure 4H). Furthermore, we performed rescue experiments to analyze the regulatory relationships among FOXP3, HDAC2, and NRF2. First, we confirmed the high efficacy of *NRF2* knockdown ( Figure 4I) and found that the increased mRNA and protein levels of HDAC2 caused by FOXP3 overexpression were reversed by NRF2 deletion ( Figure 4J). To validate the effect of TA on the FOXP3/NRF2 axis, we downregulated FOXP3 expression with specific siRNAs in DEX-treated HEI-OC1 cells ( Figure 4K, left panel) and examined NRF2 mRNA and protein levels. RT-qPCR and western blot analysis showed that TA treatment increased NRF2 mRNA and protein levels, which were decreased again by silencing of *FOXP3* ( Figure 4K, right panel and Figure 4L). In summary, TA increases FOXP3 expression, which activates NRF2-mediated HDAC2 transcription to upregulate HDAC2 expression.

### FOXP3 alleviates LPS-induced cell apoptosis via HDAC2

A previous study indicated that TA increases FOXP3 expression to inhibit the progression of nasopharyngeal carcinoma [28]. We found that FOXP3 mRNA and protein levels were significantly elevated in HEI-OC1 cells treated with LPS, followed by DEX and TA ( *P*  < 0.01; Figure 4A). To verify the regulatory effect of the FOXP3/HDAC2 axis in LPS-treated HEI-OC1 cells, we conducted rescue experiments.

**Figure 5:**
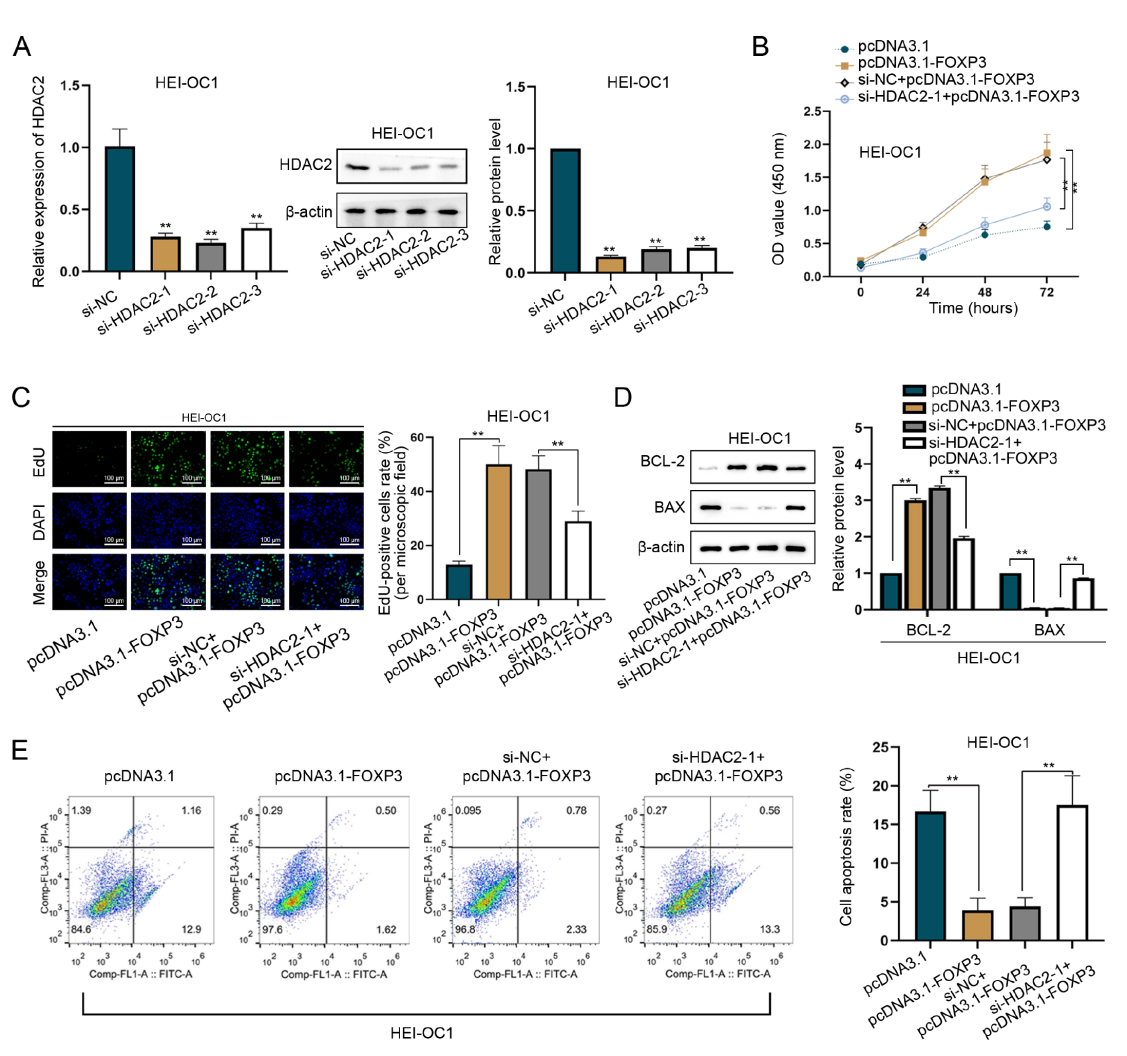
Figure 5 FOXP3 alleviates LPS-induced apoptosis via HDAC2 (A) The interference efficiency of si-HDAC2 plasmids in LPS-induced HEI-OC1 cells was determined via RT-qPCR and western blot analysis. The si-HDAC2-1 plasmid was used in subsequent experiments because it had the highest interference efficiency. (B,C) CCK-8 and EdU assays were performed to assess cell proliferation. Scale bar: 100 μm. HDAC2 knockdown abrogated the promoting effect of FOXP3 overexpression on cell proliferation. (D,E) Western blot analysis of BAX and BCL-2 expressions and flow cytometry analysis were performed to evaluate apoptosis. Elevated BCL-2 protein level and decreased BAX protein level resulted from FOXP3 upregulation were reversed by HDAC2 deficiency. Moreover, the reduction in the percentage of apoptotic cells caused by FOXP3 overexpression was reversed by HDAC2 knockdown. **P < 0.01.

First, we knocked down *HDAC2* by transfecting cells with si-HDAC2. Among the siRNAs tested, the si-HDAC2-1 plasmid had the highest interference efficiency and was used in subsequent experiments ( Figure 5A). *HDAC2* knockdown with si-HDAC2-1 abrogated the proliferative effect of FOXP3 overexpression on HEI-OC1 cells ( Figure 5B,C). The elevated BCL-2 protein level and decreased BAX protein level resulted from FOXP3 overexpression were reversed by HDAC2 deficiency ( Figure 5D). Moreover, the reduction in the cell apoptosis rate caused by FOXP3 overexpression was reversed by *HDAC2* knockdown ( Figure 5E). In summary, FOXP3 overexpression impedes LPS-induced apoptosis in HEI-OC1 cells by upregulating HDAC2 level.

## Discussion

SSHL is a prevalent emergency in otolaryngologic clinics. While GC is the first-line treatment for SSHL worldwide, resistance to GCs in many SSHL patients remains a clinical challenge [30]. Recent studies have shown that the expression of HDAC2 can increase sensitivity to GC treatment by affecting the deacetylation of glucocorticoid receptors. Reduced HDAC2 expression is considered to be an important cause of GC resistance in SSNHL [31]. HDAC2 activity is reduced through oxidative stress-induced posttranscriptional modifications, such as nitration of tyrosine residues and phosphorylation of serine residues, leading to resistance to GCs in SSNHL patients [32]. To address drug resistance, the combination of traditional Chinese medicine with Western medicine has been explored. For example, a combination therapy of GCs and breviscapine has been shown to be more effective than GCs alone for SSHL patients [12].

Previous reports have demonstrated the therapeutic value of TAs in treating various diseases. Subedi and Gaire suggested TA as an appealing drug candidate for treating neurodegenerative diseases [33]. Xue *et al*. [34] reported that TA could repress the proliferation and migration of human colon cancer cells. Although TAs exhibit antitumor activity by inducing autophagy and apoptosis and inhibit cell growth and migration by activating AMPK and inhibiting the PI3K/Akt/mTOR signaling pathway, TAs attenuate cell death in LPS- and hydrogen peroxide-induced inflammation models through the NF-κB pathway [35]. The different effects in different models may be caused by different cell types, drug concentrations, and molecular targets of the drug. In the present study, we explored the effects of TAs on the proliferation and apoptosis of cochlear cells treated with LPS as an *in vitro* SSHL model. Consistent with our previous studies [20], LPS inhibited proliferation and increased apoptosis in HEI-OC1 cells. DEX treatment significantly promoted the proliferation and inhibited the apoptosis induced by LPS. Additional TA treatment significantly enhanced the effects of DEX on proliferation and apoptosis. Our molecular mechanism study revealed that TA upregulates HDAC2 expression by activating NRF2-mediated transcription of HDAC2 through FOXP3. The predicted NRF2 binding site in the *HDAC2* promoter sequence is located at bases 419–429 (ATGACACTCCA).

HDAC2 is a critical regulator of the cell cycle, apoptosis, proliferation, migration, and differentiation [36]. Recent advancements have highlighted the role of HDAC2 in SSHL. For example, Hou *et al*. [19] proposed that reduced HDAC2 protein level may be critical for corticosteroid insensitivity in patients with refractory SSHL. Additionally, NRF2 is a transcription factor involved in mitochondrial biogenesis, autophagy, metabolic reprogramming, immunity, and inflammation [37]. Low expression levels of NRF2/HDAC2 proteins are linked to GC insensitivity in SSHL [ 10, 19] . Compared with previous studies, our current study further investigated the mechanisms underlying the interaction of TA with NRF2/HDAC2 in an *in vitro* SSHL model. This study demonstrated that TA upregulates HDAC2 expression through the activation of NRF2-mediated HDAC2 transcription. Additionally, we confirmed that TA upregulates FOXP3 expression, which in turn activates NRF2 transcription. The predicted FOXP3-binding site is located at bases 864–870 (GCAAACA) in the *NRF2* promoter sequence.

The results of our *in vitro* experiments demonstrated that TA upregulates FOXP3 expression to activate the transcription of NRF2. NRF2, in turn, promotes HDAC2 transcription, leading to increased HDAC2 expression. Elevated HDAC2 level promotes the transcriptional regulatory effect of the GR-GC complex and subsequently increases the response of cells to GCs [ 9, 19] . This molecular mechanism suggested that TA could be a potential therapeutic agent to overcome GC resistance in SSHL patients.

However, our current results are based on *in vitro* experiments using HEI-OC1 cells. In future studies, the protective effect of TA + DEX combination therapy on SSHL should be further verified in mouse models and zebrafish models to provide more comprehensive and reliable experimental support for clinical treatment. The effects of TA treatment should be further validated in the *in vivo* studies before it can be considered for use in SSHL patients.

In summary, the results of the present study demonstrated that TA can synergistically enhance the effects of GC on the proliferation and apoptosis of HEI-OC1 cells through NRF2, HDAC2 and FOXP3, indicating that TA may have therapeutic potential to ameliorate GC resistance in SSHL patients.

## References

[1] Chandrasekhar SS, Tsai Do BS, Schwartz SR, Bontempo LJ, Faucett EA, Finestone SA, Hollingsworth DB (2019). Clinical practice guideline: sudden hearing loss (update). Otolaryngol Neck Surg.

[2] Young YH (2020). Contemporary review of the causes and differential diagnosis of sudden sensorineural hearing loss. Int J Audiology.

[3] Tsuzuki N, Wasano K, Oishi N, Hentona K, Shimanuki M, Nishiyama T, Hiraga Y (2021). Severe sudden sensorineural hearing loss related to risk of stroke and atherosclerosis. Sci Rep.

[4] Kalinec GM, Park C, Thein P, Kalinec F (2016). Working with auditory HEI-OC1 cells. J Vis Exp.

[5] Taves MD, Ashwell JD (2021). Glucocorticoids in T cell development, differentiation and function. Nat Rev Immunol.

[6] Han A, Olsen O, D’Souza C, Shan J, Zhao F, Yanolatos J, Hovhannisyan Z (2021). Development of novel glucocorticoids for use in antibody–drug conjugates for the treatment of inflammatory diseases. J Med Chem.

[7] Hapgood JP, Avenant C, Moliki JM (2016). Glucocorticoid-independent modulation of GR activity: implications for immunotherapy. Pharmacol Ther.

[8] Plontke SK, Girndt M, Meisner C, Böselt I, Ludwig-Kraus B, Richter M, Rahne T (2022). Efficacy and safety of systemic, high-dose glucocorticoid therapy for idiopathic sudden sensorineural hearing loss. HNO.

[9] Zhang X, Chen J, Gao Z, Qi H, Dai Y, She W (2019). Response of glucocorticoid receptor alpha and histone deacetylase 2 to glucocorticoid treatment predicts the prognosis of sudden sensorineural hearing loss. Clin Exp Otorhinolaryngol.

[10] Qi H, Gao ZW, Hou J, Zhou Q, Ma W, Dai YH, She WD (2021). Nuclear factor erythroid 2-related factor 2-histone deacetylase 2 pathway in the pathogenesis of refractory sudden sensorineural hearing loss and glucocorticoid resistance. ORL J Otorhinolaryngol Relat Spec.

[11] Sui X, Xie T (2020). Combination of chinese and western medicine to prevent and reverse resistance of cancer cells to anticancer drugs. Chin J Integr Med.

[12] Zheng Z, Shen Y, Xia L, Wu H, Zhou H, Tang X, Meng L (2020). Glucocorticoid and breviscapine combination therapy versus glucocorticoid alone on sudden sensorineural hearing loss in patients with different audiometric curves. Adv Ther.

[13] Shi MJ, Dong BS, Yang WN, Su SB, Zhang H (2019). Preventive and therapeutic role of tanshinone IIA in hepatology. Biomed Pharmacother.

[14] Guo R, Li L, Su J, Li S, Duncan SE, Liu Z, Fan G (2020). Pharmacological activity and mechanism of tanshinone IIA in related diseases. Drug Des Devel Ther.

[15] Fang Z‐, Zhang M, Liu J, Zhao X, Zhang Y, Fang L (2021). Tanshinone IIA: a review of its anticancer effects. Front Pharmacol.

[16] Cao Y, Wang S, Li X, Zhang Y, Qiao Y (2018). The anticancer mechanism investigation of tanshinone IIA by pharmacological clustering in protein network. BMC Syst Biol.

[17] Ding B, Lin C, Liu Q, He Y, Ruganzu JB, Jin H, Peng X (2020). Tanshinone IIA attenuates neuroinflammation via inhibiting RAGE/NF-κB signaling pathway
*in vivo* and
*in vitro*. J Neuroinflamm.

[18] Monroe JD, Johnston AM, Smith ME (2020). The monofunctional platinum(II) compounds, phenanthriplatin and pyriplatin, modulate apoptosis signaling pathways in HEI-OC1 auditory hybridoma cells. NeuroToxicology.

[19] Hou J, She W, Du X, Dai Y, Xie L, Zhou Q (2016). Histone deacetylase 2 in sudden sensorineural hearing loss patients in response to intratympanic methylprednisolone perfusion. Otolaryngol Head Neck Surg.

[20] Xie L, Zhou Q, Chen X, Du X, Liu Z, Fei B, Hou J (2021). Elucidation of the Hdac2/Sp1/miR-204-5p/Bcl-2 axis as a modulator of cochlear apoptosis via
*in vivo*/
*in vitro* models of acute hearing loss. Mol Ther Nucleic Acids.

[21] Li J, Liu D, Wu J, Zhang D, Cheng B, Zhang Y, Yin Z (2016). Ginsenoside Rg1 attenuates ultraviolet B-induced glucocortisides resistance in keratinocytes via Nrf2/HDAC2 signalling. Sci Rep.

[22] Zhang S, Wu W, Jiao G, Li C, Liu H (2018). MiR-455-3p activates Nrf2/ARE signaling via HDAC2 and protects osteoblasts from oxidative stress. Int J Biol Macromol.

[23] Yang S, Liu Y, Li MY, Ng CSH, Yang S, Wang S, Zou C (2017). FOXP3 promotes tumor growth and metastasis by activating Wnt/β-catenin signaling pathway and EMT in non-small cell lung cancer. Mol Cancer.

[24] Zhu W, She W, Gao Z, Ma Y, Jin X (2022). Inhibition of macrophage migration inhibitory factor alleviates LPS-induced inflammation response of HEI-OC1 cells via suppressing NF-κB signaling. Cytokine.

[25] Bai X, Chen S, Xu K, Jin Y, Niu X, Xie L, Qiu Y (2021). N-acetylcysteine combined with dexamethasone treatment improves sudden sensorineural hearing loss and attenuates hair cell death caused by ROS stress. Front Cell Dev Biol.

[26] Liu Y, Tu H, Zhang L, Xiong J, Li L (2021). FOXP3‑induced LINC00885 promotes the proliferation and invasion of cervical cancer cells. Mol Med Rep.

[27] Tonelli C, Chio IIC, Tuveson DA (2018). Transcriptional regulation by Nrf2. Antioxid Redox Signal.

[28] Wang Y, Jin W, Wang J (2021). Tanshinone IIA regulates microRNA‑125b/foxp3/caspase‑1 signaling and inhibits cell viability of nasopharyngeal carcinoma. Mol Med Rep.

[29] Wu L, Yi B, Wei S, Rao D, He Y, Naik G, Bae S (2019). Loss of FOXP3 and TSC1 accelerates prostate cancer progression through synergistic transcriptional and posttranslational regulation of c-MYC. Cancer Res.

[30] Zhu WY, Jin X, Ma YC, Liu ZB (2020). Correlations of MIF polymorphism and serum levels of MIF with glucocorticoid sensitivity of sudden sensorineural hearing loss. J Int Med Res.

[31] Chen X, Zhang Q, Yang C, Liu Y, Li L (2021). GRβ regulates glucocorticoid resistance in sudden sensorineural hearing loss. Curr Pharm Biotechnol.

[32] Teraoka M, Hato N, Inufusa H, You F (2024). Role of oxidative stress in sensorineural hearing loss. Int J Mol Sci.

[33] Subedi L, Gaire BP (2021). Tanshinone IIA: a phytochemical as a promising drug candidate for neurodegenerative diseases. Pharmacol Res.

[34] Xue J, Jin X, Wan X, Yin X, Fang M, Liu T, Zhao S (2019). Effects and mechanism of tanshinone II A in proliferation, apoptosis, and migration of human colon cancer cells. Med Sci Monit.

[35] Yang L, Zhou G, Liu J, Song J, Zhang Z, Huang Q, Wei F (2021). Tanshinone I and tanshinone IIA/B attenuate LPS-induced mastitis via regulating the NF-κB. Biomed Pharmacother.

[36] Jo H, Shim K, Kim HU, Jung HS, Jeoung D (2023). HDAC2 as a target for developing anti-cancer drugs. Comput Struct Biotechnol J.

[37] He F, Ru X, Wen T (2020). NRF2, a transcription factor for stress response and beyond. Int J Mol Sci.

